# Spindle Cell Melanoma Presenting as an Ulcer in a Black Diabetic

**DOI:** 10.1155/2020/3083195

**Published:** 2020-10-11

**Authors:** D. A. Gaskin, D. Brathwaite, N. Depeiza, P. S. Gaskin, J. Ward

**Affiliations:** ^1^Faculty of Medical Sciences, University of the West Indies Cave Hill, Barbados; ^2^The Maria Holder Diabetes Centre for the Caribbean, Warrens, St. Michael, Barbados; ^3^Faculty of Medical Sciences, University of the West Indies Mona, Jamaica; ^4^Faculty of Science and Technology, University of the West Indies Cave Hill, Barbados

## Abstract

*Background*. Melanoma in blacks is uncommon and exceedingly rare in association with a diabetic ulcer. We present a case of a spindle cell melanoma masquerading as a diabetic ulcer. *Case Report*. A 57-year-old overweight woman presented to The Maria Holder Diabetes Centre for the Caribbean with a nonhealing ulcer of the right heel after being treated by various primary care physicians over the preceding year. Her general and systematic examinations were unremarkable. There was a 1 × 1.5 cm ulcer with a necrotic base which bled easily on contact with no evidence of peripheral neuropathy nor arterial insufficiency. Microscopic examination of a biopsy of the lesion showed fascicles of spindle cells with plump nuclei and intracytoplasmic yellow-brown pigment. Immunohistochemistry confirmed a diagnosis of melanoma. *Discussion*. There should be a high index of suspicion of malignancy with nonhealing diabetic ulcer especially when coupled with short disease duration. This case highlights the importance of a biopsy and histological evaluation in ulcers presenting in recently diagnosed diabetics with no evidence of peripheral neuropathy or vascular disease. Melanoma should be considered in spindle cell lesions especially with pigment and residual nevus cells.

## 1. Introduction

Barbados has a high prevalence of diabetes mellitus [1], and the country has been referred to as the amputation capital of the world [2]. Sumpio et al. described an increasing number of amputations associated with diabetes over a decade [3]. Despite the high prevalence of limb amputations, anecdotally, there are infrequent reports of malignancy associated with a diabetic ulcer in this setting. A search of the local literature revealed no published articles on this subject. Melanoma represents a malignant tumor which exhibits evidence of melanocytic differentiation that can be identified histopathologically or ultrastructurally. Melanocytes are melanin-producing cells that originate from the neural crest and are generally situated in the basal layer of the epidermis but can also occur at different parts of the epidermis [4]. The most common histopathologic types of melanoma are superficial spreading melanoma (70%), nodular melanoma, lentigo maligna melanoma, and acral lentiginous melanoma (2-8%). Spindle cell melanoma constitutes a relatively uncommon variant of melanoma, characterized by tumor cells with distinctly spindled morphology. Melanomas occurring on the extremities of blacks tend to be of the acral lentiginous type; however, other subtypes are much rarer [5].

Immunohistochemical stains play a vital role in the diagnosis of amelanotic, spindle cell, and epithelioid variants of melanoma and also permit distinction from poorly differentiated carcinomas as well as mesenchymal tumors. A classic case of melanoma is immunoreactive for S-100 protein, HMB-45, Melan-A, tyrosinase, Microphthalmia Transcription Factor (MITF), and vimentin. S-100 protein is a highly sensitive marker for melanomas but lacks specificity. It is positive (both nuclear and cytoplasmic staining) in 94-100% of primary and metastatic tumors [6].

We present the case of a 57-year-old black woman with a spindle cell melanoma of the right heel masquerading as a diabetic ulcer.

## 2. Case Presentation

### 2.1. Clinical History

**Figure 1:**
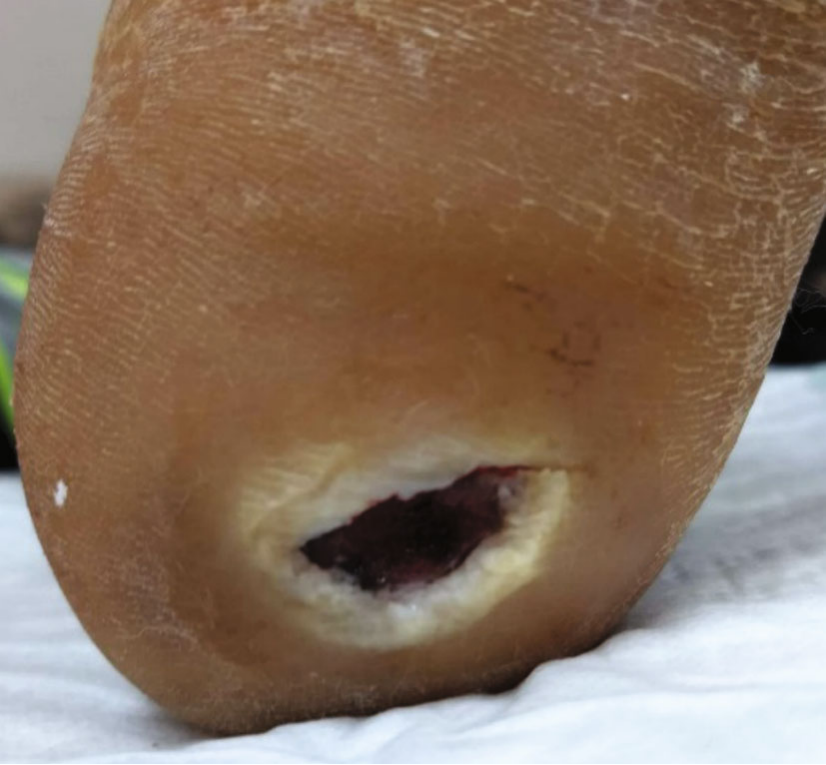
This photograph shows the ulcer located on the plantar surface of the right heel measuring 1 × 1.5 cm. The edges of the ulcer were raised, and the base was necrotic and bled easily on contact.

A 57-year-old overweight woman (BMI 28.5) with a history of type II diabetes for approximately 2 years presented to The Maria Holder Diabetes Centre for the Caribbean (Diabetes Center) with an estimated 1-year history of a nonhealing ulcer of the right heel. She reported noticing the injury after stepping on a stone, was seen by a series of primary care physicians, and subsequently was referred to the Diabetes Center because of persistence of the wound. The care in the primary setting included debridement. The original debridement sample was not examined by a pathologist. Presumably, at the time, there was no clinical suspicion of melanoma. Her general and systematic examinations were unremarkable except for uterine fibroids. There was a 1 × 1.5 cm ulcer on the right heel with a necrotic base which bled easily on contact. There was no evidence of peripheral neuropathy nor arterial insufficiency with strong biphasic to triphasic pulses. Her laboratory studies full blood count, erythrocyte sedimentation rate (ESR), and urea and electrolytes as well as liver function tests were all within the normal limits except for the haemoglobin A1c (HbA1c) which was 12. In August 2019, an area of hyperpigmentation was noted at the edge of the ulcer. By September 2019, multiple dark spots were noted in the ulcer margin (Figure 1). The wound was again debrided and the tissue sent for histology.

### 2.2. Pathology

The specimen was placed immediately in formaldehyde and sent for pathological evaluation. Macroscopic examination showed two pieces of tan-brown tissue measuring 1 × 0.3 × 0.2 cm and 0.4 × 0.3 × 0.1 cm. The tissue was paraffin-embedded, and the sections were stained with haematoxylin and eosin (H&E).

**Figure 2:**
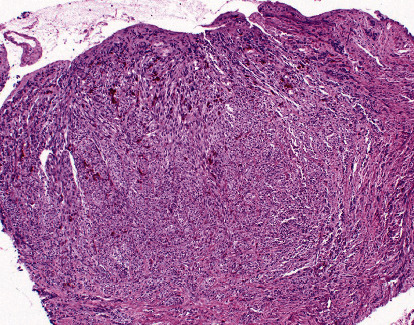
A section of the dermis from the right heel showing fascicles of focally pigmented spindle cells (H&E ×200).

Microscopic examination of the H&E-stained sections showed portions of tissue infiltrated by fascicles of spindle cells with plump nuclei and intracytoplasmic yellow-brown pigment. Mitotic figures were not identified, but nests of nevus cells were noted adjacent to the lesion (Figure 2). There was no overlying epidermis or dermis identified in the histologic sections. The differential diagnoses based on the H&E sections included melanoma, dermatofibrosarcoma protuberans (Bednar's tumor), and fibromatosis.

**Figure 3:**
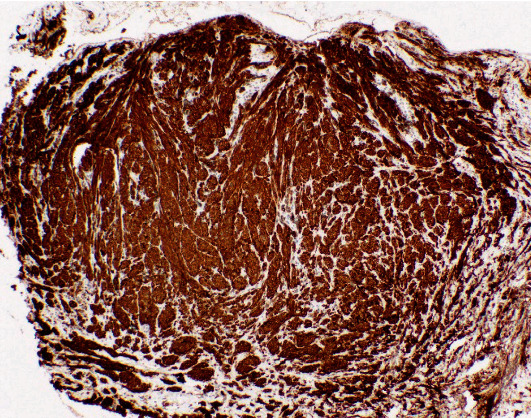
Pan melanoma markers of the right heel biopsy.

**Figure 4:**
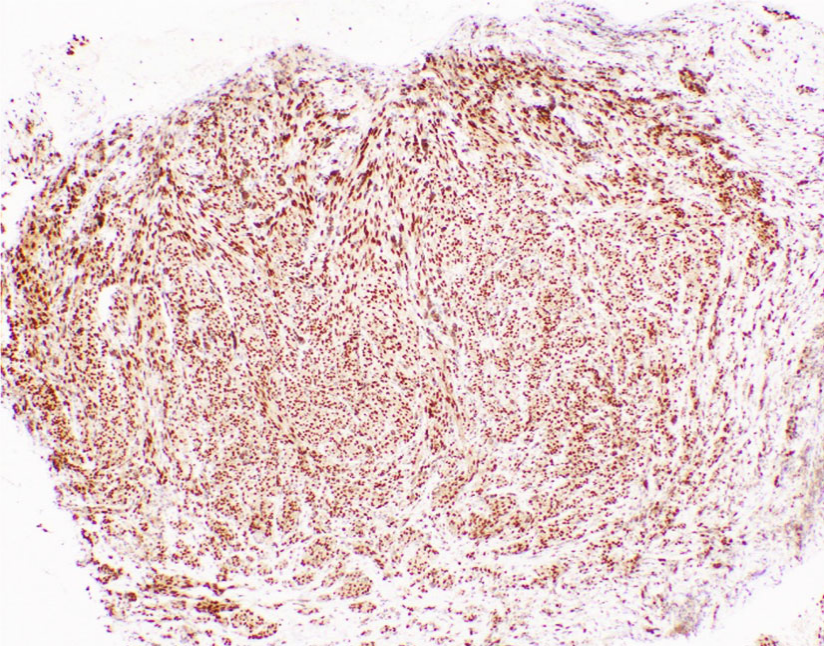
SOX10 immunohistochemical staining of the right heel biopsy showing strong nuclear staining.

Immunohistochemistry (IHC) revealed strong positivity for HMB-45, Melan-A, S-100 protein, MITF (pan melanoma markers shown in Figure 3), and SRY-Box Transcription Factor 10 (SOX10) (Figure 4) consistent with malignant melanoma (spindle cell variant).

The H&E slides were reviewed after the IHC was done; however, due to the absence of overlying epidermis, the sections could not be oriented and thus prevented the evaluation of Breslow depth or Clark's anatomical level for this tumor.

### 2.3. Clinical Follow-Up

The patient subsequently had an excision of the ulcer. However, histopathological assessment did not reveal any residual tumor. No formal lymph node dissection was performed, and the patient had no evidence of local recurrence or distant spread. No systemic therapy was administered.

## 3. Discussion

The prevalence of type II diabetes in Barbados is high [1] such that a greater occurrence of rare phenomena associated with this disease is more likely to present than in the setting with lower prevalence. Acral lentiginous melanomas are more common in peoples of African origin usually presenting on the palms and soles [7]. Most malignancies associated with diabetic ulcers are squamous cell carcinomas, representing malignant transformation of the ulcer [8]. Notably, the current case most likely represents an ulcerating melanoma and not a malignant melanoma arising in a diabetic ulcer due to the lack of peripheral neuropathy, vascular disease, and chronicity of the wound. Since diabetics are prone to ulcers, ulcerating malignancies can go undetected for extended periods. The average duration of the cancerous change growth, from the time of skin damage to malignant transformation, is in excess of 30 years [8]. This case highlights the need to consider a biopsy early in management, where the clinical features are not characteristic of the usual presentation of nonhealing ulcers. Also, consideration should be given to histopathological assessment of debridement samples in diabetic ulcers with atypical features. Clinical suspicion should be heightened by a nonhealing ulcer, particularly one that does not improve, as well as the presence of a plantar ulcer in the absence of neuropathy.

There are several reports of acral melanoma masquerading as a diabetic ulcer [9–12], and these are frequently misdiagnosed because of the atypical clinical presentation [13]. This then leads to prolonged courses of unsatisfactory therapy which leads to the possibility of disease progression.

There must be a high index of suspicion for melanocytic neoplasia when the histology shows spindle cell proliferations with pigment associated with residual nevus cells [14]. Diagnosis of melanoma is enhanced by use of Clark's levels and Breslow depth [15]. Our exploration of the current case was limited by the absence of overlying epidermis and no underlying dermis. In addition, the specimen was fragmented. The follow-up revealed no evidence of residual disease on reexcision of the biopsy site. This might be explained by the short course of disease which likely correlated to a small lesion that may have been completely removed at first excision. Ki-67 would have added to the information on the proliferative activity of the tumor but was not done as it was not required by the protocol of the College of American Pathologists.

## 4. Conclusion

This case highlights the importance of a biopsy and histological evaluation in ulcers presenting in recently diagnosed diabetics with no evidence of peripheral neuropathy or vascular disease. Pathologically, melanoma should be considered in spindle cell lesions especially with pigment and residual nevus cells.
